# Impact of pre-transplant chemotherapy cycles on allogeneic hematopoietic cell transplant outcomes in pediatric acute myeloid leukemia in first complete remission

**DOI:** 10.3389/fonc.2026.1881569

**Published:** 2026-07-29

**Authors:** Aaron Ackerman, Christen L. Ebens, Troy C. Lund, Margaret L. MacMillan, Robin Williams, Aswin Jayaraaman Chandrasekaran, Ryan Shanley, Elizabeth Krieger, Alex Hoover

**Affiliations:** 1 Department of Pediatrics, University of Minnesota, Minneapolis, MN, United States; 2 Division of Pediatric Blood and Marrow Transplantation & Cellular Therapy, Department of Pediatrics, University of Minnesota, Minneapolis, MN, United States; 3 Division of Pediatric Hematology and Oncology, Department of Pediatrics, University of Minnesota, Minneapolis, MN, United States; 4 Biostatistics Core, Masonic Cancer Center, University of Minnesota, Minneapolis, MN, United States; 5 Department of Pediatrics, Virginia Commonwealth University, Richmond, VA, United States

**Keywords:** AML, chemotherapy, HCT, pediatric, transplant

## Abstract

**Background/aims:**

Optimal pre-transplant chemotherapy exposure for pediatric patients with high-risk acute myeloid leukemia (AML) in first complete remission (CR1) remains undefined. Many patients achieve measurable residual disease (MRD)-negative remission early, raising the question of whether additional chemotherapy prior to allogeneic hematopoietic cell transplantation (HCT) improves outcomes. We evaluated the impact of pre-HCT chemotherapy cycles on post-transplant outcomes.

**Methods:**

We conducted a retrospective single-center cohort study of pediatric patients (age 0–18 years) with high-risk AML undergoing first myeloablative allogeneic HCT in MRD-negative CR1 between 2011 and 2023. Patients were stratified by receipt of 1–2 versus 3–4 pre-HCT chemotherapy cycles. Patients in the 3–4 cycle cohort who had achieved MRD-negative remission by the end of cycle 2 were assessed and compared to the 1–2 cycle cohort for rates of two-year relapse-free survival (RFS) and cumulative incidence of relapse. All patients in both cohorts were assessed for rates of two-year overall survival (OS) and one-year non-relapse mortality (NRM).

**Results:**

Forty patients met inclusion criteria; 22 (55%) received 1–2 cycles and 18 (45%) received 3–4 cycles. Excluding patients who were not MRD-negative after 2 cycles, two-year RFS was 66% (95% CI 32-86) for 3–4 cycles vs 68% (45-83) for 1–2 cycles (Hazard Ratio [HR] 0.88 [95% CI 0.26-3.01], p=0.84). The cumulative incidence of relapse at two years was 34% (9-62) in the 3–4 cycle cohort and 14% (3-31) in the 1–2 cycle cohort (HR 2.39 [0.57-10.1], p=0.23). Two-year OS was 77% (49-91) for 3–4 cycles vs 67% (46-84) in the 1–2 cycle cohort (HR 0.63 [0.18-2.14], p=0.46). One-year NRM was 0% in the 3–4 cycle group and 18% (5-37) in the 1–2 cohort (HR NE, p=0.06).

**Conclusions:**

Among pediatric patients with high-risk AML undergoing allogeneic HCT in MRD-negative CR1 at a single center, 3–4 pre-transplant chemotherapy cycles were not associated with statistically significant differences in overall or relapse-free survival compared to 1–2 cycles, although the study was underpowered to exclude clinically meaningful differences. Consolidation with HCT after 1–2 cycles of chemotherapy, once MRD-negative remission is achieved, warrants prospective exploration of risk- and remission-guided treatment strategies.

## Introduction

Outcomes for children with acute myeloid leukemia (AML), including rates of relapse-free survival (RFS) and overall survival (OS), have improved substantially, driven by therapeutic optimization and advances in disease measurement and cytogenetic and molecular risk stratification (1–3). These disease-profiling technologies, alongside data from sequential Children’s Oncology Group (COG) trials, have enabled clinicians to better define and identify high-risk disease (4, 5).

Despite progress in risk stratification and tailored approaches, outcomes for patients with high-risk disease remain poor with 5-year OS of 46% (1). Contemporary recommendations from the Children’s Oncology Group and American Society of Transplantation and Cellular Therapy for high-risk AML in pediatric patients include intensive upfront chemotherapy followed by consolidative allogeneic hematopoietic cell transplantation (HCT) (6–8). The most recent COG phase III study in upfront AML therapy, AAML1831, recommends two cycles of induction cytarabine and anthracycline-based chemotherapy, with one dose of gemtuzumab ozogamicin in first induction, followed by at least one cycle of high-dose cytarabine intensification therapy prior to HCT for high-risk patients (6, 9). Many individuals, however, achieve first complete remission (CR1) without measurable residual disease (MRD) by flow cytometry after the first or second cycle of intensive therapy, raising the question of whether additional consolidative cycles prior to HCT impact relapse, survival and transplant-related toxicity. No prior pediatric AML HCT studies have evaluated the impact of pre-HCT chemotherapy cycles after early MRD-negative remission.

Therapy-associated morbidity, resulting from anthracycline-induced cardiotoxicity, infectious complications, and other organ toxicities, contributes to both non-relapse mortality (NRM) and long-term survivorship in AML (6, 10). Reduction in chemotherapy exposure prior to HCT may reduce NRM and improve survival. We hypothesized that more cycles of pre-HCT high-intensity chemotherapy in patients transplanted in CR1 would not be associated with improved post-transplant relapse-free survival (RFS) but potentially with increased NRM.

## Methods

This retrospective, single-center cohort study included pediatric patients (age 0-18) diagnosed with high-risk acute myeloid leukemia based on cytogenetics or refractory disease status who underwent first allogeneic myeloablative HCT in CR1 between 2011 and 2023 at the University of Minnesota (UMN) Masonic Children’s Hospital. In accordance with the Declaration of Helsinki, all parents/guardians gave consent for the IRB-approved UMN Blood and Marrow Transplantation (BMT)/Cell Therapy (CT) Database from which the data for this manuscript were extracted. All patients were required to have a bone marrow assessment with aspirate multiparameter flow cytometry after their final cycle of chemotherapy, within 30 days prior to preparative therapy for HCT per institutional standards. Multiparameter flow cytometry was performed consistently at either at patients’ local institutions or at the University of Minnesota clinical laboratory. Patients with MRD positivity defined as >0.05% by flow cytometry, those in CR2 or greater, those who received a reduced-intensity conditioning and patients with Down syndrome were excluded.

Baseline demographic characteristics, disease burden, treatment history, laboratory values and HCT outcomes data were retrospectively analyzed. Kaplan-Meier estimates of survival (and RFS) were computed and compared using the log-rank test. Confidence intervals were calculated using a log-log transformation. Cumulative incidences of relapse at two years and non-relapse mortality (NRM) at one year were estimated using the cumulative incidence function, with the other event defined as a competing risk, and compared using Gray’s test. One-year NRM was selected *a priori* because deaths occurring beyond the first post-transplant year are increasingly influenced by chronic GVHD and late transplant complications rather than factors directly related to pre-transplant chemotherapy exposure. Relapse in the RFS and cumulative incidence of relapse analyses was defined by morphologic recurrence of AML after HCT and NRM was defined as death occurring without prior relapse. Additional analysis was descriptive. R software version 4.4.2 was used for analysis.

## Results

**Figure 1:**
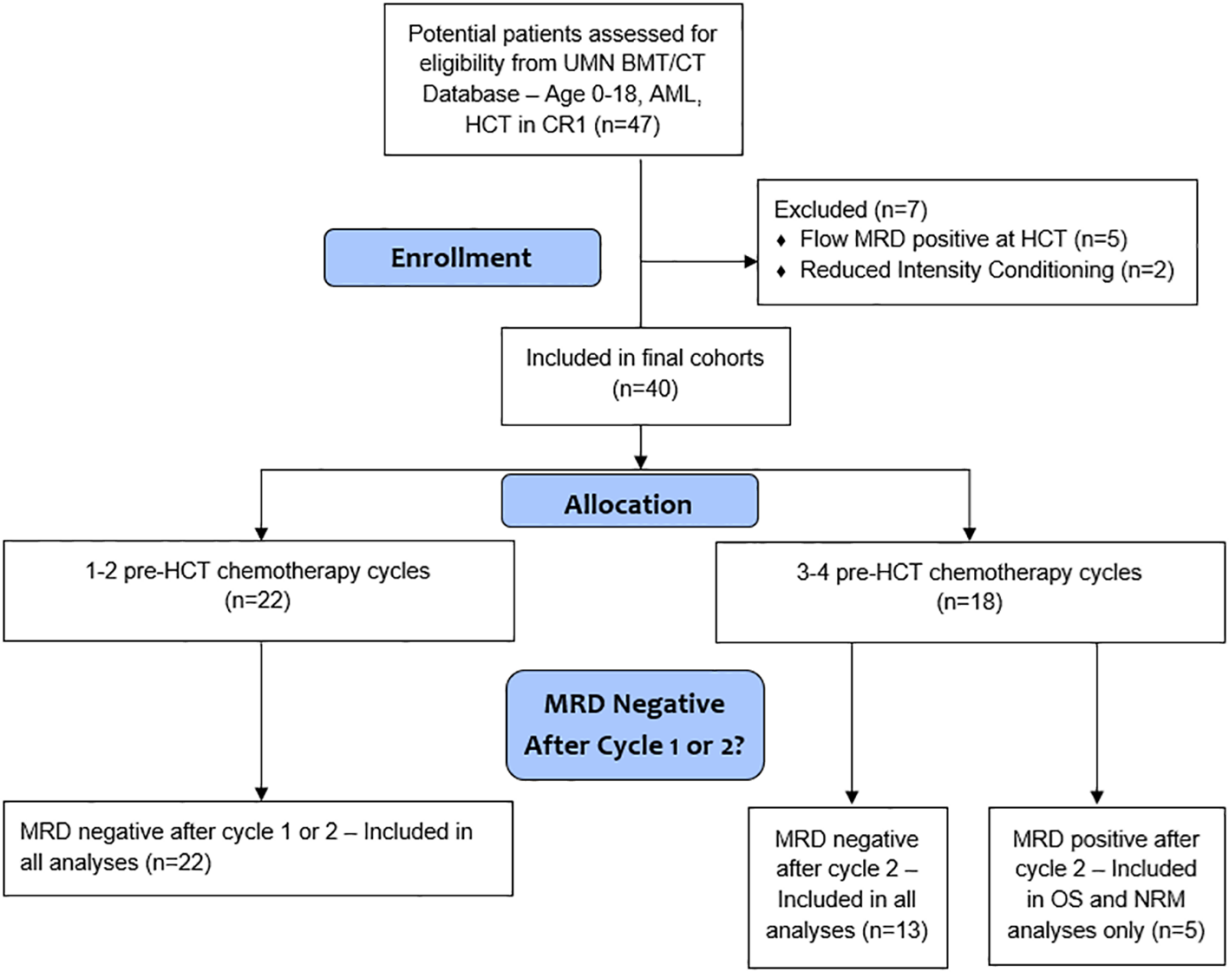
Cohort selection flow diagram.

Forty patients met the inclusion criteria, as shown in the cohort selection flow diagram (**Figure 1**). All patients received cytarabine-based intensive chemotherapy cycles to achieve CR prior to transplant. Median age at HCT was 8.8 years (Range 0.4-18.7 years). All patients were conditioned with busulfan- (n=27) or total body irradiation (TBI)-based (n=13) preparative regimens, and all received a calcineurin inhibitor-based graft-vs-host disease (GVHD) prophylaxis, with 6 patients (15%) also receiving post-transplant cyclophosphamide (PTCy). Twenty-two patients (55%) received 1–2 cycles of chemotherapy prior to HCT, 18 (45%) received 3–4 cycles. Of the 18 patients that received 3–4 cycles, 13 patients were in MRD-negative remission after 2 cycles but went on to receive additional cycles prior to HCT; this sub-cohort was utilized for the RFS and relapse comparative analyses with the 1–2 cycle cohort. No patients in this subgroup had morphologic relapse, MRD recurrence or clinical progression prior to HCT. Age, sex, race/ethnicity, Karnofsky/Lansky score, AML cytogenetics, extramedullary disease presence, donor type, stem cell source, days from diagnosis to HCT, conditioning regimen, ATG exposure, and GVHD prophylaxis are shown in **Table 1**. Upfront disease treatment and response details including MRD status at end-of-induction 1, cycles of chemotherapy to morphologic CR1, cycles of chemotherapy to MRD negativity by flow cytometry, gemtuzumab exposure during cycle 1 or 2, and total cycles prior to HCT are shown in **Table 2**.

**Table 1:** Demographic, disease and transplant characteristics.

Characteristic	Overall N = 40*^1^*	1–2 cycles N = 22*^1^*	3–4 cycles (early MRD negative) N = 13*^1^*	3–4 cycles (late MRD negative) N = 5*^1^*
Age [Median (Q1, Q3)]	8.8 (2.5, 14.0)	11.8 (4.9, 14.1)	5.8 (2.6, 9.3)	5.0 (1.6, 13.9)
Sex				
Female	20 (50%)	12 (55%)	6 (46%)	2 (40%)
Male	20 (50%)	10 (45%)	7 (54%)	3 (60%)
Race				
African American	3 (7.5%)	2 (9.1%)	0 (0%)	1 (20%)
American Indian	1 (2.5%)	0 (0%)	0 (0%)	1 (20%)
Asian	3 (7.5%)	2 (9.1%)	1 (7.7%)	0 (0%)
Caucasian	30 (75%)	16 (73%)	11 (85%)	3 (60%)
Mixed	1 (2.5%)	1 (4.5%)	0 (0%)	0 (0%)
Unknown	2 (5.0%)	1 (4.5%)	1 (7.7%)	0 (0%)
Karnofsky/Lansky Score				
100	16 (40%)	6 (27%)	6 (46%)	4 (80%)
90	9 (23%)	6 (27%)	2 (15%)	1 (20%)
80	14 (35%)	9 (41%)	5 (38%)	0 (0%)
70	1 (2.5%)	1 (4.5%)	0 (0%)	0 (0%)
AML Cytogenetic Grouping				
CEBPA	1 (2.5%)	0 (0%)	1 (7.7%)	0 (0%)
Del(5q)	1 (2.5%)	1 (4.5%)	0 (0%)	0 (0%)
ETV6	3 (7.5%)	1 (4.5%)	2 (15%)	0 (0%)
FLT3/ITD	8 (20%)	5 (23%)	3 (23%)	0 (0%)
KMT2A	18 (45%)	10 (45%)	6 (46%)	2 (40%)
MLLT10	1 (2.5%)	0 (0%)	0 (0%)	1 (20%)
Monosomy 7	1 (2.5%)	1 (4.5%)	0 (0%)	0 (0%)
None	2 (5.0%)	1 (4.5%)	0 (0%)	1 (20%)
NUP98	5 (13%)	3 (14%)	1 (7.7%)	1 (20%)
Extramedullary involvement any time prior to HCT				
Yes	12 (30%)	3 (14%)	6 (46%)	3 (60%)
No	25 (63%)	17 (77%)	6 (46%)	2 (40%)
Unknown	3 (7.5%)	2 (9.1%)	1 (7.7%)	0 (0%)
Donor				
Matched Sibling	12 (30%)	4 (18%)	4 (31%)	4 (80%)
Double UCB	5 (13%)	4 (18%)	1 (7.7%)	0 (0%)
Single UCB	21 (53%)	13 (59%)	7 (54%)	1 (20%)
URD	2 (5.0%)	1 (4.5%)	1 (7.7%)	0 (0%)
HLA Matching				
8/8	7 (18%)	4 (19%)	2 (15%)	1 (20%)
6/6	10 (25%)	3 (14%)	3 (23%)	4 (80%)
5/6	8 (20%)	5 (23%)	3 (23%)	0 (0%)
4/6	10 (25%)	6 (28%)	4 (31%)	0 (0%)
4/6 + 4/6	1 (2.5%)	0 (0%)	1 (7.7%)	0 (0%)
4/6 + 5/6	1 (2.5%)	1 (4.5%)	0 (0%)	0 (0%)
5/6 + 5/6	1 (2.5%)	1 (4.5%)	0 (0%)	0 (0%)
5/6 + 6/6	2 (5.0%)	2 (9.1%)	0 (0%)	0 (0%)
Days from Diagnosis to Transplant	125 (99, 153)	100 (96, 122)	139 (130, 174)	139 (123, 163)
Stem cell source				
Cord Blood	26 (65%)	17 (77%)	8 (62%)	1 (20%)
Bone Marrow	12 (30%)	4 (18%)	4 (31%)	4 (80%)
Peripheral Blood	2 (5.0%)	1 (4.5%)	1 (7.7%)	0 (0%)
Conditioning agents				
Busulfan/Fludarabine +/- ATG	7 (17.5%)	4 (18%)	2 (15%)	1 (20%)
Busulfan/Melphalan/Fludarabine	10 (25%)	5 (23%)	4 (31%)	1 (20%)
Busulfan/Thiotepa/Fludarabine/ATG	6 (15%)	5 (23%)	1 (7.7%)	0 (0%)
Busulfan/Cyclophosphamide	3 (7.5%)	1 (4.5%)	1 (7.7%)	1 (20%)
Busulfan/Cyclophosphamide/Fludarabine	1 (2.5%)	1 (4.5%)	0 (0%)	0 (0%)
TBI/Cyclophosphamide	6 (15%)	1 (4.5%)	3 (23%)	2 (40%)
TBI/Cyclophosphamide/Fludarabine	7 (18%)	5 (23%)	2 (15%)	0 (0%)
ATG?	8 (20%)	6 (27%)	2 (15%)	0 (0%)
GVHD prophylaxis agents				
CNI/MMF	26 (65%)	17 (77%)	8 (62%)	1 (20%)
CNI/MTX	8 (20%)	1 (4.5%)	4 (31%)	3 (60%)
PTCy/Tac/MMF	6 (15%)	4 (18%)	1 (7.7%)	1 (20%)

*^1^*Median (Q1, Q3); n (%)

MRD, measurable residual disease (positivity defined as >0.05% leukemic cells by flow cytometry); Del, deletion; UCB, umbilical cord blood; URD, unrelated donor; ATG, anti-thymocyte globulin; TBI, total body irradiation; GVHD, graft-versus-host disease; CNI, calcineurin inhibitor; MMF, mycophenolate mofetil; MTX, methotrexate; PTCy, post-transplant cyclophosphamide; Tac, tacrolimus.

**Table 2:** Disease response to upfront chemotherapy.

Characteristic	Overall N = 40*^1^*	1–2 cycles N = 22*^1^*	3–4 cycles (early MRD negative) N = 13*^1^*	3–4 cycles (late MRD negative) N = 5*^1^*
EOI1 MRD positive (>0.05% by flow)				
Yes	17 (43%)	10 (45%)	3 (23%)	4 (80%)
No	22 (55%)	12 (55%)	10 (77%)	0 (0%)
Unknown	1 (2.5%)	0 (0%)	0 (0%)	1 (20%)
Cycles to CR1 (<5% blasts by morph)				
1	27 (68%)	14 (64%)	10 (77%)	3 (60%)
2	11 (28%)	8 (36%)	3 (23%)	0 (0%)
3	1 (2.5%)	0 (0%)	0 (0%)	1 (20%)
4	1 (2.5%)	0 (0%)	0 (0%)	1 (20%)
Cycles to MRD neg (<0.05% by flow)				
1	23 (58%)	13 (59%)	10 (77%)	0 (0%)
2	12 (30%)	9 (41%)	3 (23%)	0 (0%)
3	3 (7.5%)	0 (0%)	0 (0%)	3 (60%)
4	1 (2.5%)	0 (0%)	0 (0%)	1 (20%)
Unknown	1 (2.5%)	0 (0%)	0 (0%)	1 (20%)
Gemtuzumab during cycle 1 or 2	10 (25%)	7 (32%)	2 (15%)	1 (20%)
Total cycles prior to HCT				
1	6 (15%)	6 (27%)	0 (0%)	0 (0%)
2	16 (40%)	16 (73%)	0 (0%)	0 (0%)
3	16 (40%)	0 (0%)	12 (92%)	4 (80%)
4	2 (5.0%)	0 (0%)	1 (7.7%)	1 (20%)

*^1^*n (%)

MRD, measurable residual disease; EOI1, end of induction 1; CR1, first complete remission; morph, bone marrow morphology; flow, multiparameterflow cytometry. MRD positivity was defined as >0.05% leukemic cells by flow cytometry. Morphologic complete remission (CR1) was defined as <5% bone marrow blasts.

In order to limit the potential bias of persistent/refractory disease on relapse outcomes, only the thirteen patients in the 3–4 cycle cohort that had reached MRD-negative remission by the end of cycle 2 are included in RFS and relapse analyses. This subset will thus be referred to as the 3–4 cycle early MRD-negative subgroup.

The cohorts were relatively similar in cytogenetic mutation profile with KMT2A rearrangements in 10 patients (45%) in the 1–2 cycle cohort and 8 (44%) in the 3–4 cycle full cohort. Fourteen of the 1–2 cycle patients (64%) had reached morphologic CR1 after one cycle of chemotherapy, 13 (59%) of whom were MRD-negative. Median duration from diagnosis to transplant was 100 days (IQR 96, 122) in the 1–2 cycle cohort, 139 (130, 174) in the 3–4 cycle early MRD-negative subgroup and 139 (123, 163) in the 3–4 cycle late MRD-negative subgroup.

**Figure 2:**
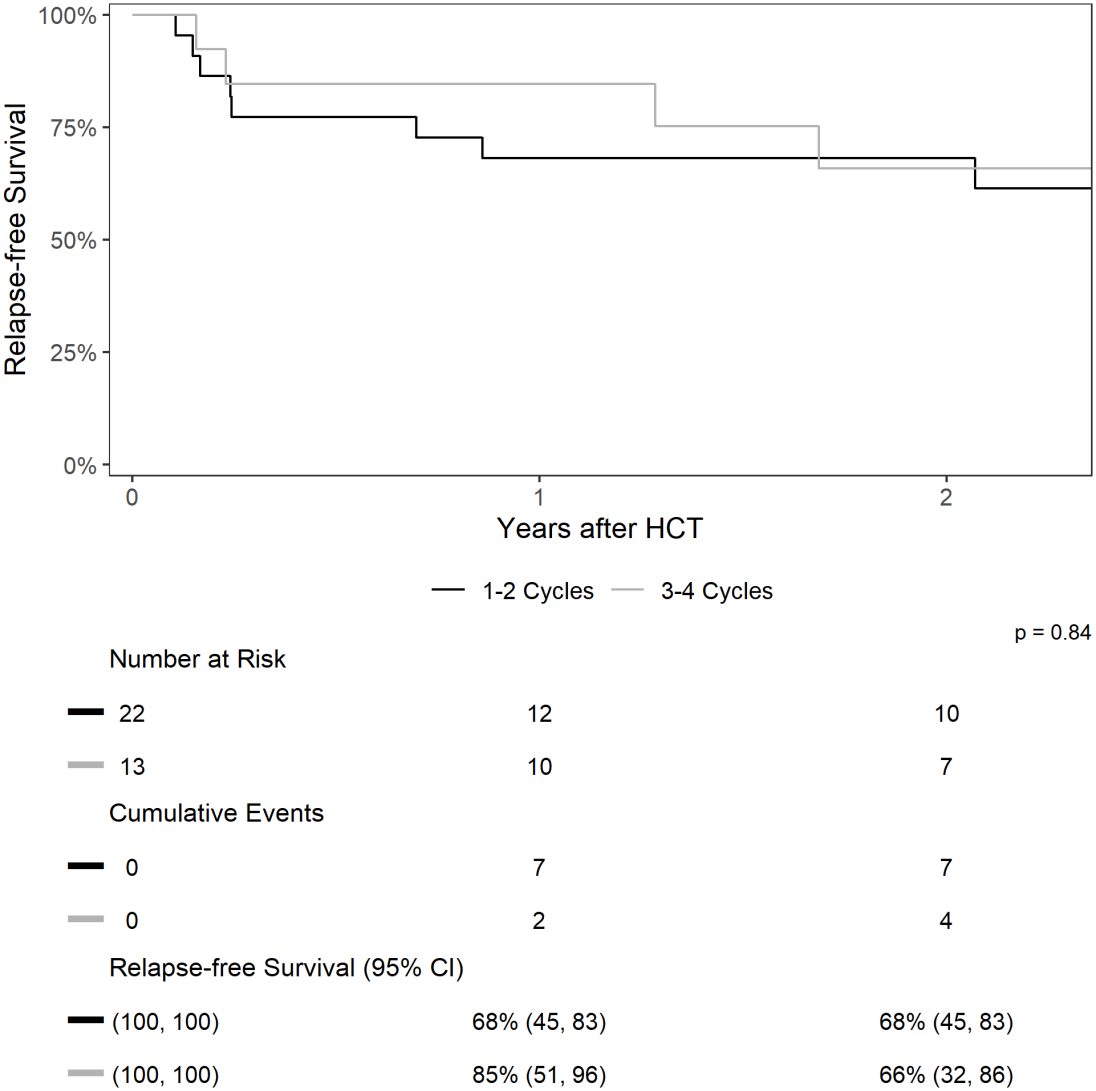
Relapse-free survival (RFS).

**Figure 3:**
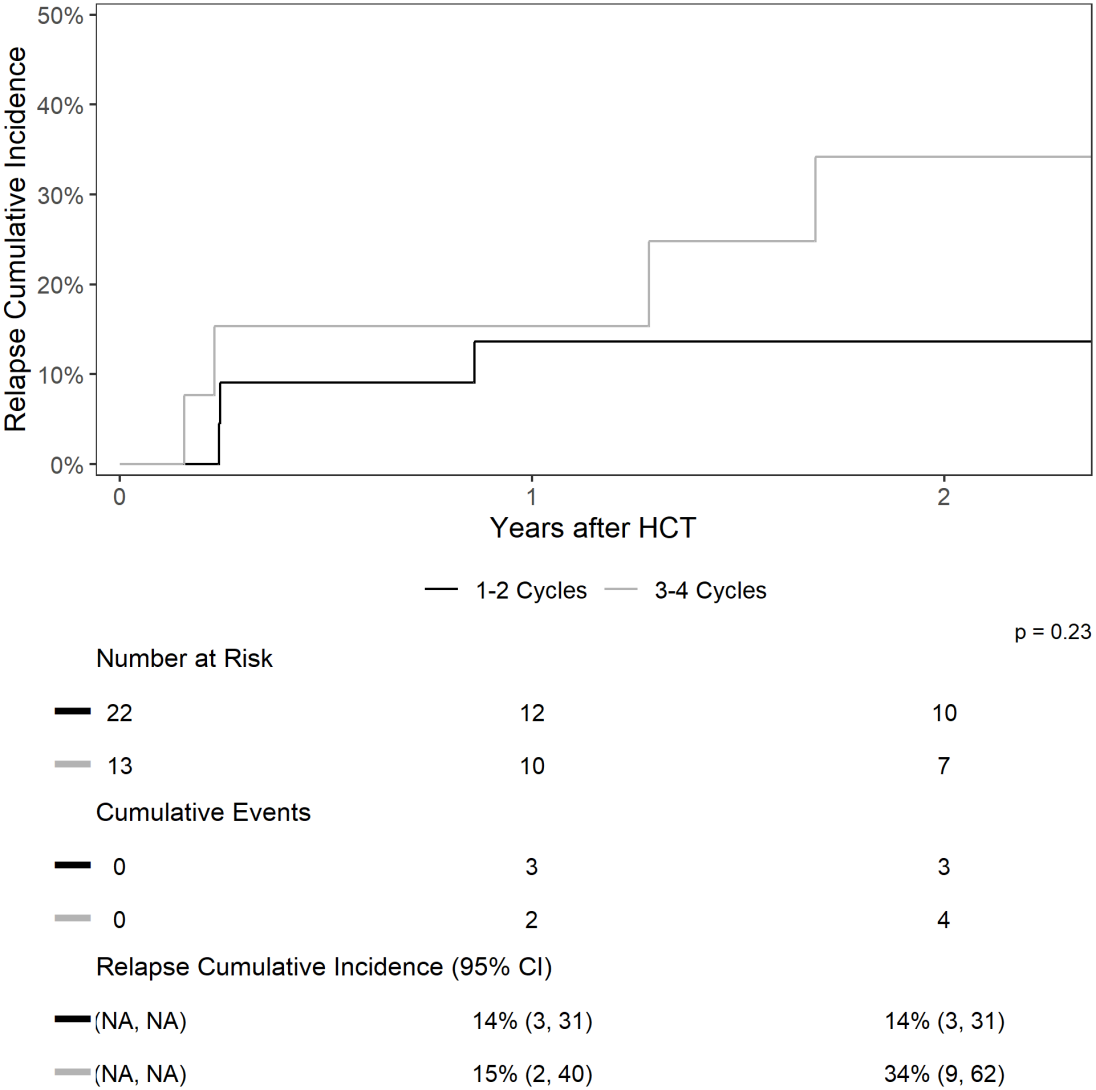
Cumulative incidence of relapse.

Two-year RFS was 68% (95% CI 45-83) in the 1–2 cycle cohort and 66% (32-86) in the 3–4 cycle early MRD-negative subgroup (**Figure 2**); risk of relapse or death was not significantly different between cohorts with hazard ratio (HR) of 0.88 (0.26-3.01; p = 0.84) for the 3–4 cycle early MRD-negative subgroup relative to the 1–2 cycle cohort. Cumulative incidence of relapse at 2 years was 14% (3-31) in the 1–2 cycle cohort vs 34% (9-62) in the 3–4 cycle early MRD-negative subgroup (**Figure 3**) with HR for relapse in this subgroup of 2.39 (0.57-10.1), p = 0.23.

**Figure 4:**
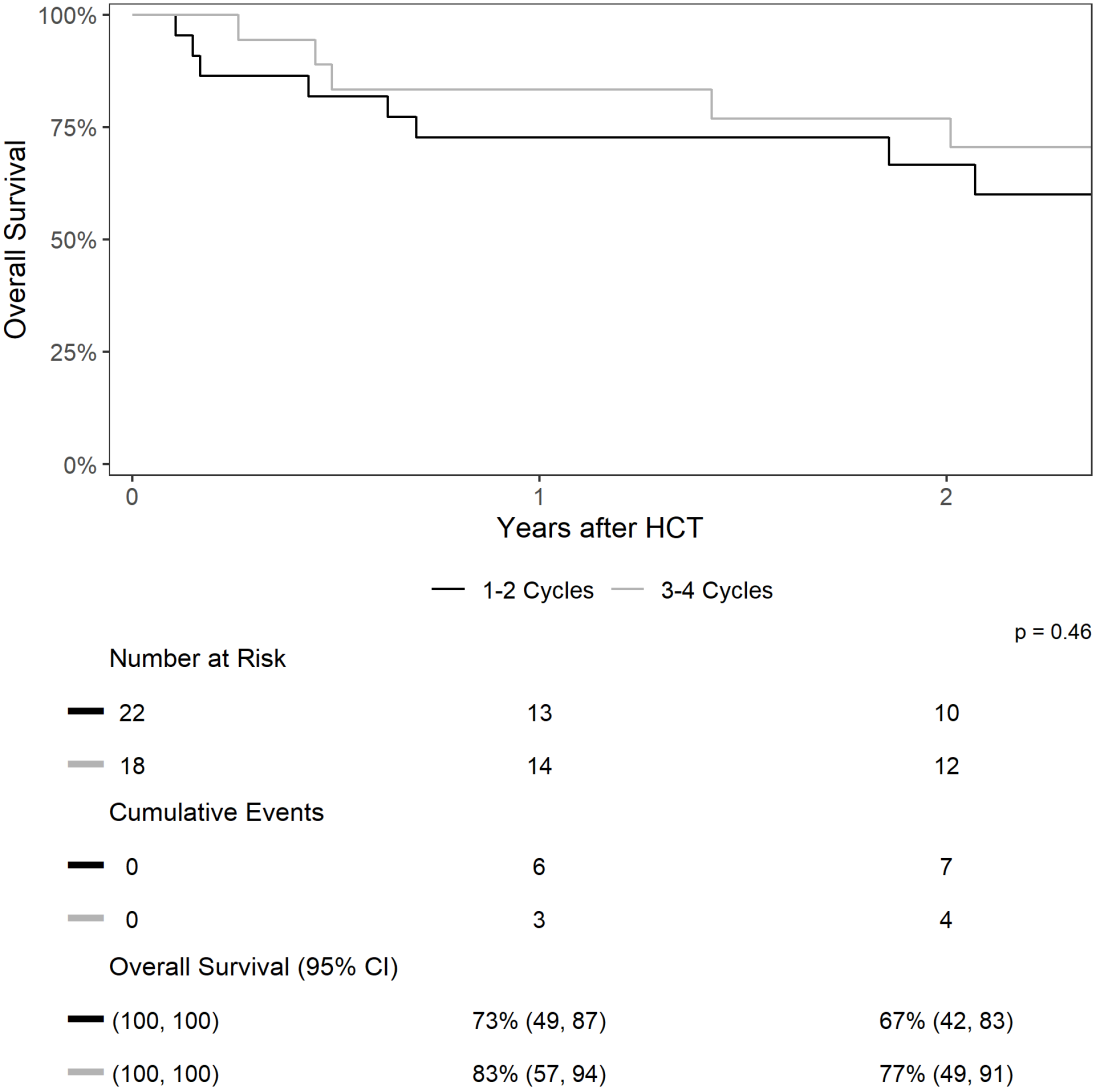
Overall survival (OS).

**Figure 5:**
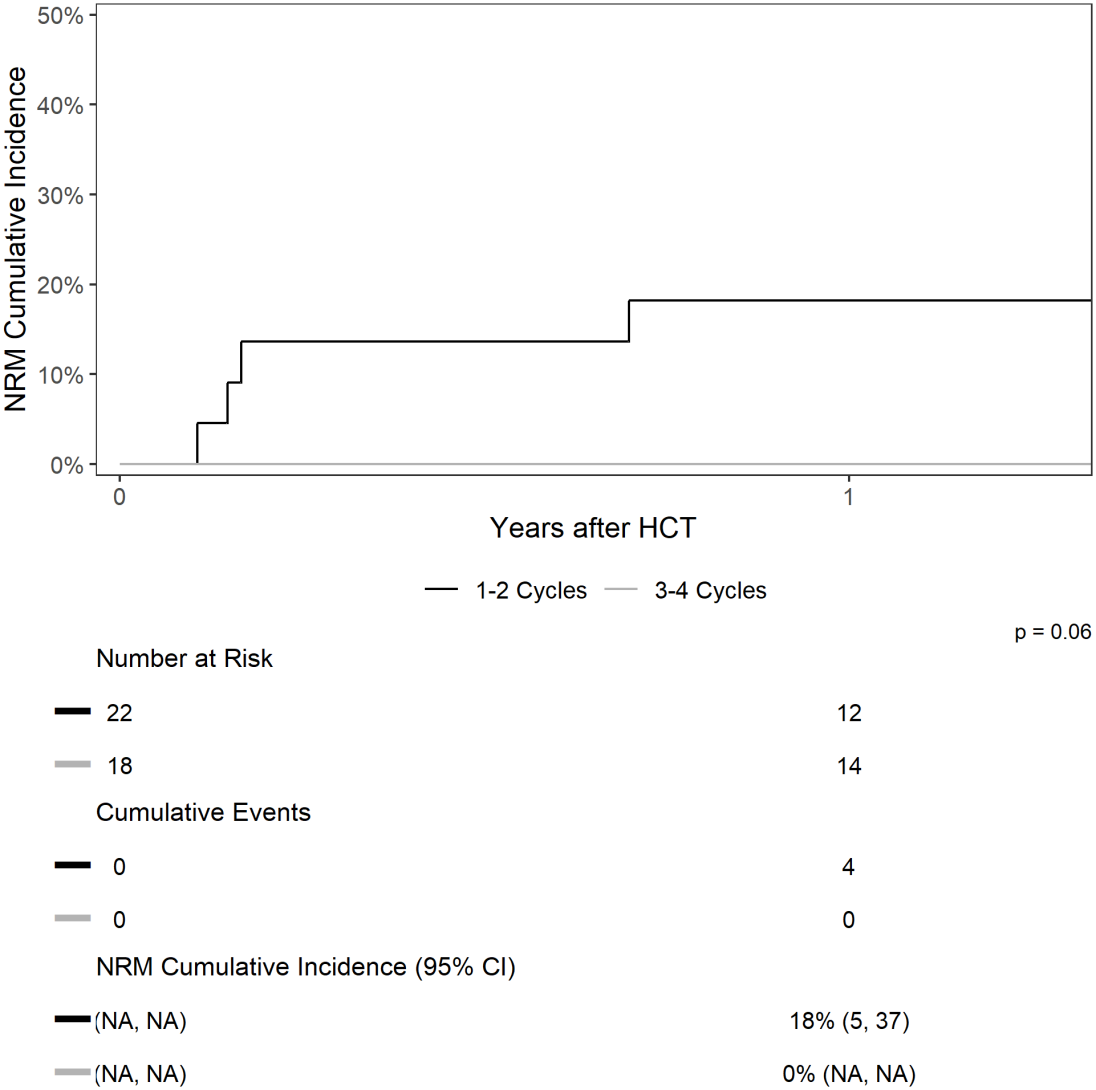
Non-relapse mortality (NRM).

All patients in the 3–4 cycle cohorts were included in the overall survival and non-relapse mortality analyses. Two-year OS was 67% (42-83) in the 1–2 cycle cohort and 77% (49-91) in the 3–4 cycle cohort (**Figure 4**) with HR for death in the 3–4 cycle cohort of 0.63 (0.18-2.14), p = 0.46. The 1–2 cycle cohort had a one-year NRM incidence of 18% (5-37); there was no NRM in the 3–4 cycle full cohort so HR was non-evaluable, p=0.06 (**Figure 5**). Comparative analyses are shown in **Table 3**. The four NRM events in the 1–2 cycle cohort were attributable to idiopathic pneumonia syndrome (n=2), diffuse alveolar hemorrhage with cerebral edema (n=1), and diffuse alveolar hemorrhage with CMV pneumonitis (n=1).

**Table 3:** Comparative cohort analyses.

Outcome	N events by year 2 (year 1 for NRM)	Hazard Ratio	95% CI	P value
Overall Survival (OS)	11	0.63	(0.18, 2.14)	0.46
Relapse Free Survival* (RFS)	11	0.88	(0.26, 3.01)	0.84
Cumulative Incidence of Relapse*	7	2.39	(0.57, 10.1)	0.23
Non-Relapse Mortality (NRM)	4	NE	NE	0.06

Hazard ratios are for 3–4 cycles relative to 1–2 cycles. RFS and Relapse analyses compare 3–4 cycle early MRD-negative subgroup to 1–2 cycle cohort.

NE, not estimable.

## Discussion

In this retrospective cohort, a reduced number of pre-transplant chemotherapy cycles for pediatric patients with high-risk AML who had achieved CR1 was not associated with inferior transplant outcomes. To our knowledge, this is the first analysis to assess whether fewer cytarabine-based chemotherapy cycles prior to allogeneic HCT affect transplant outcomes. Additional pre-HCT cycles beyond MRD-negative remission showed no significant differences in RFS or OS. Although not statistically significant, additional cycles were associated with a higher 2-year relapse rate (34% vs 14%), but lower rates of non-relapse mortality (0% vs 18%). These findings suggest that once an MRD-negative remission is attained, proceeding directly to transplant without additional chemotherapy was not associated with worse disease control or survival; however, the study was underpowered to exclude clinically meaningful differences in relapse, survival, or NRM.

The aim of this study was to explore a crucial gap currently in the literature. To date, no studies have addressed whether patients already MRD-negative after 1 or 2 intensive cytarabine-based chemotherapy cycles could safely omit additional cycles before transplant. Likewise, studies have clearly demonstrated that consolidative HCT in CR1 improves survival in high-risk AML (7), but investigators did not evaluate the optimal number of pre-transplant cycles. Contemporary pediatric AML protocols have included multiple cycles of intensive chemotherapy before HCT based on historical data regarding the importance of high-dose cytarabine (11). For example, the current COG trial AAML1831 mandates two induction courses plus at least one high-dose cytarabine intensification before HCT for high-risk patients, and earlier studies (e.g., AAML0531) employed multiple consolidation cycles with gemtuzumab ozogamicin to reduce relapse (1). Patients in this retrospective study primarily received therapy either on study or according to the active upfront AML COG protocols during their respective times of treatment (AAML0531, AAML1031 and AAML1831).

Several factors may explain why additional cycles failed to improve relapse risk after HCT. In the upfront setting, intensive chemotherapy confers significant toxicity (cardiotoxicity, severe infections) that can increase mortality (6). Predictably, in our cohort, patients receiving 3–4 cycles had a longer time interval from diagnosis to transplant (median 139 vs 100 days), which may allow time for leukemia progression, outgrowth of a chemotherapy-resistant leukemia clone or increase treatment-related complications. Although the cohorts were quite similar in demographics, disease characteristics and transplant approach, a few potentially impactful differences between the cohorts included a lower median age in the 3–4 cycle cohort – 5.8 years vs 11.8 years – higher rates of extramedullary disease at any point prior to HCT in the 3–4 cycle cohort – 50% vs 14% – and more matched sibling donor transplants in the 3–4 cycle cohort – 44% vs 18%.

Notably, 72% (13/18) of patients who received 3–4 cycles had already achieved MRD-negative remission by the second cycle and were thus included in the RFS and relapse analyses. Given the similar rates of RFS and nominally higher relapse rate in the 3–4 cycle cohort, once MRD negativity is attained, residual leukemia may be below the threshold for additional chemotherapeutic benefit and better addressed by transplant conditioning and graft-versus-leukemia effects. Correspondingly, a COG analysis comparing outcomes from AAML1031 with the prior upfront study, AAML0531, showed that intensifying the second induction cycle did not improve survival but did increase toxicity (10).

Non-relapse mortality was substantial in the 1–2 cycle cohort with 4 deaths in 22 treated patients. NRM cause of death was idiopathic pneumonia syndrome in 2 patients, diffuse alveolar hemorrhage (DAH) and cerebral edema in one patient, and DAH with cytomegalovirus (CMV) pneumonitis in another patient. Interestingly, there was no NRM in the 3–4 cycle cohort, contradicting the theory that increased chemotherapy exposure increases the likelihood of transplant-related toxicity. Differences in transplant platform and donor source may have contributed to the observed NRM imbalance between cohorts, however absence of NRM events in the 3–4 cycle cohort should be interpreted cautiously given the small sample size, competing risks, and possible selection bias. We focused on 1-year NRM as a prespecified endpoint because deaths occurring later after HCT are increasingly driven by chronic GVHD and other late transplant-related complications rather than factors directly related to pre-transplant chemotherapy burden. Consequently, extending the NRM analysis to 2 years would be less informative for the specific clinical question addressed in this study.

The limitations of this study are substantial and include those inherent to a small, retrospective, single-center cohort, including limited statistical power and the potential for residual confounding in a non-randomized comparison. Treatment allocation was not protocolized and may reflect clinician preference, patient- or disease-specific factors, or unrelated donor availability which could bias observed associations. Importantly, the analysis is conditioned on having successfully proceeded to allogeneic HCT in CR1; thus, it excludes patients who never reached transplant because of refractory disease, early relapse, or, critically, treatment-related morbidity or mortality that precluded HCT. This may cause selection, or survivorship, bias and likely underestimates the real-world toxicity burden of additional chemotherapy cycles as the study design cannot estimate the full risk-benefit balance of giving additional chemotherapy from the time of MRD-negative CR, because pre-HCT toxicity and progression among non-transplanted patients are not captured. Additionally, differences in donor type, stem cell source and era of treatment corresponding with transplant-related care and other supportive care known to play a role in NRM, GVHD risk, immune reconstitution and relapse – particularly the higher proportion of cord blood grafts in the 1–2 cycle cohort and matched sibling donors in the 3–4 cycle cohort – may have influenced NRM and survival outcomes.

Prospective studies are warranted to further explore these findings. Future trials could test MRD-guided therapy reduction – for example, whether high-risk patients who reach MRD-negative CR after 1–2 cycles can proceed directly to HCT – and incorporate molecular risk stratification. Certain subgroups (e.g., KMT2A-rearranged AML) carry a high relapse risk and benefit from adjunctive targeted therapies like gemtuzumab ozogamicin or menin inhibition rather than additional chemotherapy (12–14). Similarly, patients with FLT3-ITD mutations benefit from targeted FLT3 inhibitors in combination with chemotherapy (15, 16). By leveraging early response and genetic risk indicators with validation in prospective trials, it may be possible to safely reduce chemotherapy exposure in MRD-negative pediatric AML, a strategy that could improve tolerability without sacrificing cure rates.

## Data Availability

The raw data supporting the conclusions of this article will be made available by the authors, without undue reservation.
